# Outpatient treatment of COVID-19 with steroids in the phase of mild pneumonia without the need for admission as an opportunity to modify the course of the disease: A structured summary of a randomised controlled trial

**DOI:** 10.1186/s13063-020-04575-w

**Published:** 2020-07-09

**Authors:** Miriam Saiz-Rodríguez, Teresa Peña, Lourdes Lázaro, Ángel González, Andrés Martínez, José A. Cordero, Juan T. Vicente, Fernando Richard, María Jesús Coma, Martín de Frutos, Jorge Labrador, Ana Pueyo

**Affiliations:** 1 Research Unit, Hospital Universitario de Burgos, Burgos, Spain; 2 Neumology Department, Hospital Universitario de Burgos, Avda Islas Baleares, 3, Burgos, Spain; 3 Gerencia de Atención Primaria de Burgos, Burgos, Spain; 4 Instituto de Investigación Sanitaria Bioaraba, Vitoria-Gasteiz, Álava Spain; 5 Emergency Department, Hospital Universitario de Burgos, Burgos, Spain; 6 Hematology Department, Hospital Universitario de Burgos, Burgos, Spain

**Keywords:** COVID-19, Randomised controlled trial, Protocol, Early pulmonary, Prednisone, Outpatients

## Abstract

**Objectives:**

The aim of this study is to explore the effectiveness and safety of oral corticosteroids (prednisone) in the treatment of early stage SARS-Cov-2 pneumonia in patients who do not yet meet hospital admission criteria.

**Trial design:**

Randomized clinical trial, controlled, open, parallel group, to evaluate the effectiveness of steroids in adult patients with confirmed COVID-19, with incipient pulmonary involvement, without hospital admission criteria. Patients will be stratified by the presence or not of radiological data on pneumonia.

**Participants:**

We will include patients with early stage SARS-Cov-2 pneumonia who do not meet hospital admission criteria from the reference hospital, the Hospital Universitario de Burgos, in the region of Castilla y León, Spain. Patients will be followed-up by specialist physicians and Primary Health Care professionals.

Inclusion criteria:

- Men and women.

- Age between 18 and 75 years old.

- Diagnosed SARS-CoV-2 infection, by PCR and/or IgM+ antibody test and/or antigen test.

- Clinical diagnosis of lung involvement: (respiratory symptoms +/- pathological auscultation +/- O2 desaturation)

- Chest X-ray with mild-moderate alterations or normal.

- Patients who give their verbal informed consent in front of witnesses, which will be reflected in the patients’ medical records.

Exclusion criteria:

- Oxygen desaturation below 93% or P0_2_ < 62.

- Moderate-severe dyspnea or significant respiratory or general deterioration that makes admission advisable.

- Chest X-ray with multifocal infiltrates.

- Insulin-dependent diabetes with poor control or glycaemia in the emergency room test greater than 300 mg/ml (fasting or not).

- Other significant comorbidities: Severe renal failure (creatinine clearance < 30 mL/min); cirrhosis or chronic liver disease, poorly controlled hypertension.

- Heart rhythm disturbances (including prolonged QT).

- Severe immunosuppression (HIV infection, long-term use of immunosuppressive agents); cancer.

- Pregnant or breast-feeding women.

- Patients under use of glucocorticoids for other diseases.

- History of allergy or intolerance to any of the drugs in the study (prednisone, azithromycin or hydroxychloroquine).

- Patients who took one or more of the study drugs in the 7 days prior to study inclusion.

- Patients taking non-suppressible drugs with risk of QT prolongation or significant interactions.

- Patients unwilling or unable to participate until study completion.

- Participation in another study.

**Intervention and comparator:**

Eligible patients will be randomized to receive standard outpatient treatment only (group 1) or standard outpatient treatment plus prednisone (group 2).

- Group 1: paracetamol 1 g/8 h (on demand) + hydroxychloroquine 400 mg/12h the first day, 200 mg/12 h for 4 days + azithromycin 500 mg/24h for 5 days.

- Group 2: paracetamol 1 g/8 h (on demand) + hydroxychloroquine 400 mg/12h the first day, 200 mg/12 h for 4 days + azithromycin 500 mg/24h for 5 days + prednisone 60 mg / 24 h for 3 days, 30 mg / 24 h for 3 days and 15 mg / 24 h for 3 days.

**Main outcomes:**

If the patient requires ambulatory observation, according to the protocol established in this respect in the Emergency Department, meets all the criteria for inclusion and none for exclusion, data will be taken by the person responsible on the data collection sheet. The main result is admission after 30 days. Secondary outcomes are 30-day ICU admission and hospital stay. The safety variable will be the occurrence of clinical symptoms or delirium related to the steroids. Also, the possible decompensations of diabetes will be measured. All tests will be on an intention-to-treat basis.

**Randomisation:**

Treatment will be assigned according to stratified randomization by the presence or absence of radiological data of lung involvement (previously performed by random sequence 1:1 generated with Epidat and kept hidden by opaque, sealed envelopes, which will only be opened after inclusion and basal measurement).

**Blinding (masking):**

Participants, caregivers and personnel responsible for outcomes measurement will not be blinded to group assignment, once the patient is included and the basal measurement performed, as per protocol design.

**Numbers to be randomised (sample size):**

The percentage of patients with incipient lung involvement is unknown, but given that pulmonary involvement already exists it is estimated to be around 20%. We consider that the intervention could reduce this percentage to 5%, so the necessary sample size would be 200 subjects (100 per group), with a power of 80% and an estimated loss percentage of 10%.

**Trial Status:**

The protocol with code TAC-COVID-19, version 2.0 on date: April 16, 2020 is approved by the Spanish Drug Agency (AEMPS) and the local Ethics Committee. The trial is in the recruitment phase. Recruitment began 19 April, 2020 and is anticipated to be complete by April 2021.

**Trial registration:**

The trial was registered under the title “OUTPATIENT TREATMENT OF EARLY PULMONARY COVID19 WITH CORTICOSTEROIDS AS AN OPPORTUNITY TO MODIFY THE COURSE OF THE DISEASE” with EudraCT number 2020-001622-64, registered on 3 April 2020.

**Full protocol:**

The full protocol is attached as an additional file, accessible from the Trials website (Additional file 1). In the interest in expediting dissemination of this material, the familiar formatting has been eliminated; this Letter serves as a summary of the key elements of the full protocol.

## Supplementary information

**Additional file 1.** Full Study Protocol.

## Data Availability

The data will be available from the author on reasonable request. Please contact the corresponding author Dra. Ana Pueyo (apueyo@saludcastillayleon.es).

