# Supplementary material for: Outpatient treatment of COVID-19 with steroids in the phase of mild pneumonia without the need for admission as an opportunity to modify the course of the disease: A structured summary of a randomised controlled trial
Source: Trials. 2020 Jul 9;21:632. doi: 10.1186/s13063-020-04575-w (PMC7344042; doi:10.1186/s13063-020-04575-w)
Supplement: Supplementary file 1 — Additional file 1. Full Study Protocol. [file 13063_2020_4575_MOESM1_ESM.docx]

ADDITIONAL FILE 1: STUDY PROTOCOL

**Protocol code:** TAC-COVID-19 version 2.0, 16/APR/2020

**Title:** OUTPATIENT TREATMENT OF COVID-19 WITH STEROIDS IN THE PHASE OF MILD PNEUMONIA WITHOUT THE NEED FOR ADMISSION AS AN OPPORTUNITY TO MODIFY THE COURSE OF THE DISEASE

**Sponsor:** Dr Ana Pueyo Bastida

**Principal Investigator:**

Dr Ana Pueyo Bastida

Pneumologist

Hospital Universitario de Burgos

Avda Islas Baleares, 3

09006 Burgos, Spain

email: [apueyo@saludcastillayleon.es](mailto:apueyo@saludcastillayleon.es)

EUDRACT: 2020-001622-64

Centre of execution: Hospital Universitario and and Primary Care Management of Burgos, Spain.

Study drug: prednisone

INTRODUCTION

SARS-CoV-2 triggers an abnormal immune response, which appears to play a central role in disease progression, morbidity and mortality. Despite the fact that the clinical picture is much less abrupt than in other viral processes and that the symptomatology and alterations in the physical examination precede the most serious stage by several days, in many cases, when the treatment that could be effective is prescribed, lung damage is already established. The result is a high percentage of hospital admissions, needing for ventilatory support, prolonged intensive care unit (ICU) stays, and hospital collapse, with very significant mortality.

In pneumology, from a clinical point of view, and based on many years of experience in treating respiratory diseases with an immunological response (severe asthma, exacerbations of idiopathic pulmonary fibrosis, flu with distress, etc., processes in which an agent triggers an immune response that is complex and in many aspects similar to this coronavirus disease), we feel that on this occasion we are too late.

On the other hand, Primary Care represents in most cases the first contact with the patient. Moreover, they follow-up the patient after infection diagnosis and detect the worsening on those occasions when the patient does not require admission initially. The lack of effective treatment at this stage, and the unfavourable evolution in many cases, means that the primary care physician, who should be a key player in the evolution of the picture, has tight margin for manoeuvre. The impression in both areas, Pneumology and Primary Care, is that the disease continues its course without anything being done to modify the trajectory, until we reach the final phase in which all resources, aggressive treatments, prolonged stays, etc. are necessary.

Currently, the different phases of the disease course are known and their relationship with viral activity, initially, and inflammatory response, later. The clinical, auscultation, oxygenation or radiological repercussions are demonstrative of the physiopathology and evolution. The expressiveness of the data we can obtain in a simple way is important to understand what is happening in the lung and, probably, when we should act.

We consider that there is a window of opportunity for action, prior to the need for admission, which could be used to modify the clinical course of the disease.

PROJECT RATIONALE

As demonstrated in previous epidemics of coronavirus (SARS, MERS), the protective inflammatory response that usually occurs in the face of aggression to the respiratory system can, in the case of these viruses, be dysregulated and pathogenic. In these cases, the lesion is produced at the pulmonary level, with special emphasis on macrophages and pneumocytes, leaving the airway quite protected. As the lung, besides the bone marrow, is the organ with the greatest wealth of pluripotent cells, the complex interaction response between innate and adaptive immunity, with release of cells and mediators of inflammation, is magnified. On the other hand, given the direct contact of the focus of inflammation, the alveolus, with a multitude of blood vessels, this response can transcend the rest of the body. This same fact provides very important information for the management of the disease by providing evidence in blood of the presence of cytokines and other mediators of inflammation, that can be a fundamental biological marker to direct therapies.

The efficacy of systemic steroids has been highly controversial in viral pneumonias prior to this pandemic. Numerous papers reported, as well as meta-analyses, do not reflect satisfactory results in terms of survival or hospital stay. Virtually all of them have been performed on the critical patient, intubated in ICU. A description of the coronavirus-induced alveolar lesion gives a better understanding of the lack of efficacy. Thus, the analysis of necropsies performed during the SARS epidemic in 2003 showed an important lesion, practically centered in the lung: coexistence of lung consolidation, edema, focal hemorrhages and diffuse alveolar damage with hyaline membrane formation, alveolar hemorrhage, fibrin exudation in the alveolar spaces, together with alveolar and septal fibrosis, observed in the last stages; that is, lesions with little subsidiary response to the corticoid.

A significant presence of macrophages and neutrophils was also demonstrated, which correlated with lower CD4 and CD8 LT lymphocyte levels in peripheral blood.

Neither in our environment nor globally is the generalized use of steroids recommended, and the convenience of individualizing their use and in the context of clinical trials is indicated given the absence of evidence of the convenience or not of their use. In recent weeks, however, high-dose systemic corticosteroids have become the first-line treatment at the time of hospital admission. Its use is not planned as an outpatient, even in patients with clear lung involvement. The decision whether to prescribe corticosteroids or not varies even in the same patient in a few hours, since patient’s evolution might worsen dramatically.

It is known that, in general, these drugs reduce lung inflammation but also inhibit the immune response and clearance of the pathogen, being the fear of a negative effect on viral replication the main reason for avoidance of corticosteroid prescription. In respiratory pathology, the use of preventive treatment with inhaled corticoids, evidences a fundamental role in the decrease in the morbidity and mortality of asthmatic exacerbations (including those triggered by viruses) and the modulation of the inflammatory response. There is no evidence for or against such a benefit in the case of coronaviruses, whose inflammatory effect is more focused on the lung.

The description of coronavirus behavior in patients from Wuhan, China, has allowed us to establish the clinical course of the disease, with the main mechanism involved in each phase. Thus, the first stage would be determined by the direct action of the virus in the upper airway, where it replicates. It was shown that the viral load is higher in the first week, with mild upper track and fever symptoms.

The second, often separated from the previous one by a transitional period of clinical improvement, would indicate the beginning of pulmonary involvement. The influence of the virus is much smaller, and negative PCR results at the oronasal level are not uncommon. At this stage, the situation is not serious, but there are already clinical, auscultation and, in many cases, faint data of radiological involvement, indicating the onset of lung inflammation. It would correspond to stage II, or pulmonary phase, early (IIA) and established (IIB), according to the intensity of the alterations.

Finally, stage III or hyperinflation occurs, characterized by amplification of the inflammatory response, with the so-called cytokine storm that triggers an exaggerated inflammatory response and the establishment of a very significant lung damage. This alveolar occupation by inflammatory material that prevents gas exchange, causing severe hypoxemic respiratory failure, could lead in many cases to death. In phases II and III, although of course genetic material of the virus continues to be detected, the viral load is probably not so important anymore, while the inflammatory phenomenon is. We currently do not know what triggers this exaggerated inflammatory response in some individuals, and not in others, as already described in the 2009 pandemic influenza, which revealed the existence of an inflammatory disease with different forms of immune response expression. Genetic or epigenetic variations may justify the special severity, along with different morbidity-mortality, present in different geographical areas or ethnic groups. So far, it should be considered that any infected individual, especially at the ages when the immune response is most "rich", has the potential to develop a serious or even fatal condition.

Detecting when pulmonary involvement is starting is relatively simple in a clinical way: any level of dyspnea, an abnormal pulmonary auscultation (basically some crackling, even hypoventilation with difficulty in deep inspiration, which translates into loss of elasticity by alveolar occupation). Abnormalities in O2 saturation or in gasometry must be attributed to pulmonary involvement, since we have been able to clinically verify the little bronchial involvement of the virus, unlike what happens with other frequent pathogens such as influenza or respiratory syncytial virus, in which the bronchus and patients with airway diseases are especially affected. Generally, these clinical anomalies are accompanied by radiological alterations, which are initially subtle and very evident in more advanced stages.

The fear of the use of steroids in these phases is not reflected in the facts observed in these weeks, both by our group and by pulmonologists from other communities with an even higher incidence of the disease than ours. It is striking that patients with asthma or chronic obstructive pulmonary disease (COPD), who use inhaled steroids daily and even corticodependent patients (requiring chronic treatment with oral steroids), are not being especially affected by the disease. It may even appear that they are less affected than the general population, which has suggested to us the possibility of a certain protective role for chronic inhaled treatments of these patients, which will probably generate further studies. In those who do have SARS-CoV-2 infection, the course is no worse than that of patients not receiving such treatments.

A study conducted on cultures of nose and trachea epithelial cells infected with other HCoV-229E coronaviruses suggests that glycopyronium, formoterol and a combination of both with budesonide inhibit viral replication and modulate virus-induced inflammation in the airway. In addition, a recent *in vitro* study conducted during the current pandemic suggests that the steroid ciclesonide (a steroid compound used by inhalation as a treatment for asthma) has the ability to suppress viral replication of SARS-CoV-2, which opens the possibility for the use of other types of steroids.

On the other hand, the decision to start or not the treatment with steroids at very high doses sometimes seems more related to the decision of admission than to the patient’s clinical data. The guidelines and duration of different treatments are modified daily, motivated by papers, in many cases series of cases, that describe adequate responses of some of the drugs that are being used. The availability of drugs, or the description of side effects, or the lack of effectiveness shown in some of the series also conditions these strategies.

Based on the above, we hypothesize that treatment with systemic steroids, in the early phase of pulmonary involvement, without significant established alveolar damage, could prevent or at least modulate the subsequent inflammatory response, avoiding or decreasing the severity of the condition.

These patients, in a phase that generally does not require admission, are usually treated by Primary Care, which in this case would have treatment tools beyond some drugs that act as antivirals (in a phase in which the presence of the virus is no longer so important) and antibiotics (it should be remembered that in these phases we are hardly seeing any bacterial infections, probably due to the fact that in this viriasis the bronchial mucosa is not affected, and therefore the barrier that prevents the penetration of the bacteria is maintained).

We therefore consider that there is a window of opportunity for a pharmacological action, in which treatment with oral steroids on an outpatient basis can modify the course of COVID-19.

The aim of this study is to explore the effectiveness and safety of oral steroids (prednisone) in the treatment of SARS-CoV-2 pneumonia, in early stages, in patients who do not yet meet hospital admission criteria.

MATERIALS AND METHODS

TRIAL DESIGN

Randomized clinical trial, controlled, open, parallel group, to evaluate the effectiveness of steroids in adult patients with confirmed COVID-19, with incipient pulmonary involvement, without hospital admission criteria. Patients will be stratified by the presence or not of radiological data on pneumonia.

Eligible patients will be randomized to receive prednisone plus standard therapy, or standard therapy alone. Patients in the prednisone group will receive, in addition to the rest of the established treatment, a dose of 60 mg orally, every 24 hours for 3 consecutive days, followed by 30 mg 3 days, and 15 mg 3 days.

PARTICIPANTS

We will include patients with SARS-Cov-2 infection, confirmed by PCR and/or IgM+ and/or antigen test, with clinical data and exploration compatible with pulmonary involvement, with or without data on pneumonia evident in radiology, without hospital admission criteria.

Inclusion criteria:

- Men and women.
- Age between 18 and 75 years old.
- Diagnosed SARS-CoV-2 infection, by PCR and/or IgM+ antibody test and/or antigen test.
- Clinical diagnosis of lung involvement: (respiratory symptoms +/- pathological auscultation +/- O2 desaturation)
- Chest X-ray with mild-moderate alterations or normal.
- Patients who give their verbal informed consent in front of witnesses, which will be reflected in the patients’ medical records.

Exclusion criteria:

- Desaturation below 93% or P0_2_ < 62.
- Moderate-severe dyspnea or significant respiratory or general deterioration that makes admission advisable.
- Chest X-ray with multifocal infiltrates.
- Insulin-dependent diabetes with poor control or glycaemia in the emergency room test greater than 300 mg/ml (fasting or not).
- Other significant comorbidities: Severe renal failure (creatinine clearance < 30 mL/min); cirrhosis or chronic liver disease, poorly controlled hypertension.
- Heart rhythm disturbances (including prolonged QT).
- Severe immunosuppression (HIV infection, long-term use of immunosuppressive agents); cancer.
- Pregnant or breast-feeding women.
- Patients under use of glucocorticoids for other diseases.
- History of allergy or intolerance to any of the drugs in the study (prednisone, azithromycin or hydroxychloroquine).
- Patients who took one or more of the study drugs in the 7 days prior to study inclusion.
- Patients taking non-suppressible drugs with risk of QT prolongation or significant interactions.
- Patients unwilling or unable to participate until study completion.
- Participation in another study.

PROJECT DEVELOPMENT

Patients that attend to the Emergency Department, either by their own decision or by referral from a Primary Care health professional, when they present symptoms of low respiratory tract, persistent cough, dyspnea, tightness in the chest, or for reappearance of fever, worsening or lack of improvement, after several days of previous clinic (general malaise, fever, upper tract symptoms, diarrhoea...).

Once in the Emergency Department, the established triage and action procedure will be performed, which includes confirmation of infection by PCR or determination of IgM (as established by the health authorities). Patients with a confirmed diagnosis of infection in previous days, and who have not received other treatment that is not symptomatic, will also be included. In the Emergency Department, we will perform:

- Auscultation.

- O2 saturation and/or gasometry.

- Chest x-ray.

- Blood samples, including inflammation markers and hemogram.

- Electrocardiogram (ECG).

Then, depending on the results:

1. If the patient is a subsidiary of admission, will not be included in the study.

2. If the patient requires ambulatory observation, according to the protocol established in this respect in the Emergency Department, meets all the criteria for inclusion and none for exclusion, data will be taken by the person responsible on the data collection sheet.

Treatment will be assigned according to stratified randomization by the presence or absence of radiological data of lung involvement (previously performed by random sequence 1:1 generated with Epidat and kept hidden by opaque, sealed envelopes, which will only be opened after inclusion and basal measurement).

Participants, caregivers and personnel responsible for outcomes measurement will not be blinded to group assignment, once the patient is included and the basal measurement performed, as per protocol design.

INTERVENTION AND COMPARATOR

Eligible patients will be randomized to receive standard outpatient treatment only (group 1) or standard outpatient treatment plus prednisone (group 2).

- Group 1: paracetamol 1 g/8 h (on demand) + hydroxychloroquine 400 mg/12h the first day, 200 mg/12 h for 4 days + azithromycin 500 mg/24h for 5 days.
- Group 2: paracetamol 1 g/8 h (on demand) + hydroxychloroquine 400 mg/12h the first day, 200 mg/12 h for 4 days + azithromycin 500 mg/24h for 5 days + prednisone 60 mg / 24 h for 3 days, 30 mg / 24 h for 3 days and 15 mg / 24 h for 3 days.

An updated and operational contact telephone number, as well as the reference address, will be noted for all patients for subsequent follow-up in Primary Care.

MAIN OUTCOMES

The main result is admission after 30 days. Secondary outcomes are 30-day ICU admission and hospital stay. The safety variable will be the occurrence of clinical symptoms or delirium related to the steroids. Also, the possible decompensations of diabetes will be measured. All tests will be on an intention-to-treat basis.

FOLLOW-UP

The follow-up will be performed by telephone for 2 weeks according to the protocol established in Primary Care, keeping a written copy of the whole process, and completing the actions in the case report form (CRF).

If the evolution is satisfactory, at 15 ± 2 days, auscultation and measurement of O2 saturation will be carried out, as well as ECG. That day, a new medication kit will be collected. If any medication was not administered the reason will be registered (intolerance, forgetfulness, side effects...).

Throughout the follow-up, decisions will be made to refer to the Emergency Department or modify the action, at the discretion of the physician responsible.

A chest X-ray and analysis (including inflammation markers, blood glucose and HbA1C) will be performed at one month (30 ± 3 days).

PCR (or antigen test, if applicable) will be attempted 15 days after inclusion in the study, to determine the persistence of the virus in the upper airway in relation to the treatments received.

In the event of abandonment of the study, the reason will be recorded and a final evaluation will be made. In the case of loss, the latest available information will be used and the value allocation will be assessed according to the time of loss.

SAMPLE SIZE

The percentage of patients with incipient lung involvement is unknown, but given that pulmonary involvement already exists it is estimated to be around 20%. We consider that the intervention could reduce this percentage to 5%, so the necessary sample size would be 200 subjects (100 per group), with a power of 80% and an estimated loss percentage of 10%.

SECURITY NOTIFICATION:

Those events considered adverse drug reactions, serious adverse drug reaction, or unexpected serious adverse drug reactions, as defined in Royal Decree 1090/2015 regulating clinical trials, will be notified.

STATISTICAL ANALYSIS:

The descriptive analysis of the qualitative variables will be carried out by means of frequency distributions and of the quantitative ones by means of mean and standard deviation (or median and interquartile range in case they do not fit a normal distribution).

For the analysis of the main outcome variables, both efficacy (comparison of the percentage of hospital admissions at 30 days) and safety (percentage of psychotic events related to corticosteroids), chi-square tests will be used. The same tests will be used to analyze the relationship between the rest of the qualitative variables. For the comparison of the quantitative variables, T-Student (or Mann-Whitney U will be used).

The analysis includes a sex- and stratum-specific analysis to identify possible differences in the expected results.

The analysis will be by intention to treat and will be carried out with the statistical package SPSS version 23, establishing a significance level of 0.05. Confidence intervals will be calculated at 95%.

STUDY LIMITATIONS

An upper age limit of 75 years was established, which would limit the results to this population group. On the one hand, it has been described that older patients account for the highest percentage of deaths. However, since the immune response in the elderly is not as strong, it is less frequent to see established pulmonary dysfunction, but important comorbidities, which often represent the cause of death. This fact could dilute the effect of the action. Given that the study tries to find in which patients the result of modifying current clinical practice produces the greatest benefit in terms of avoiding anomalous immune response, in order to apply it in clinical practice, we consider that this bias does not significantly modify the result.

In the phase of early pulmonary infection, the radiological manifestations are often not very perceptible (faint infiltrations, scarce aeration at the bases...) without the picture being established as frank pneumonia. A CT scan in these situations would reveal tarnished (ground) glass that is not demonstrable in simplex Rx. In the current circumstances in our environment it is not possible to perform such a study on all these patients. However, we know from what has been described and proven in our patients that a bad evolution in subsequent days is not infrequent, despite an initial normal X-ray. This group of patients without admission criteria would be precisely the most benefited if it is shown that early intervention modifies the course of the disease. To be exclusive, discarding patients who (despite other clinical or exploratory signs suggestive of pulmonary involvement) do not have radiological pneumonia would deprive them of the possible benefit of action. To avoid this problem, a randomization has been made for each stratum, depending on whether or not they have radiological signs. This will allow a more balanced distribution in the treatment arms and a subsequent analysis of results more in line with the usual clinical situation.

Another limitation is caused by the way in which the protocols of action change day by day. This means that no fixed criteria have been established for inclusion, in terms of action in the Emergency Department and the decision of admission, and therefore the establishment of complete treatment or not. Given that our aim is to determine whether the modification in usual clinical practice, including steroids in the outpatient treatment scheme, alters the course of the disease, it is left to the discretion of the Emergency Department to establish the cut-off points of the respiratory situation measures (sat O2, pO2) to decide on admission or outpatient treatment. This decision, since it does not depend on the research team, is unlikely to bias the results of the study.

Another aspect to consider is the availability of drugs. In recent weeks, we have seen how the decision to treat widely with hydroxychloroquine or azithromycin means that treatment guidelines must be modified or their duration shortened. Therefore, there is no guarantee that the treatment schedule will remain stable. However, it is very likely that it will continue to include symptomatic treatment, associated with antibiotic + / - modifier of viral replication. If, as a result of supply problems, any of these treatments are no longer provided, one group with prednisone and another without prednisone will still be maintained, allowing the role of prednisone to be further assessed.

Although at this moment the decision to prescribe one or another treatment in admitted patients is made based on biological parameters in blood, in the Emergency Department this has not been established as the basis for decisions. On the other hand, in the current situation, based on achieving greater agility in decision making and being able to initiate treatment as early as possible, we consider that clinical-radiological management is sufficient to decide on subsequent action.

Finally, although to date the diagnosis is based on the demonstration of viral PCR in nasopharyngeal samples, it is possible that the availability of IgM detection methods, or antigen detection methods, which could be carried out in Primary Care, could modify the scope of selection and randomization of patients. However, the researchers in charge of the field work in one or another setting would belong to Primary Care, and the rest of the procedure would be similar, so the validity of the study is maintained.

Based on safety criteria, in order to prevent written documents from acting as vectors for the transmission of the virus, it has been decided to collect verbal informed consent before witnesses, recording it in the electronic history, based on the recommendations of the Guidance on the Management of Clinical Trials during the COVID-19 (Coronavirus) pandemic Version 2, 27/03/2020. The written information sheet will be provided to the patient.

Although the use of questionnaires aimed at recognizing symptoms by telephone has been considered, in the follow-up of people in isolation due to infection, these questionnaires, such as the Roth, are not adequately validated, so we have finally opted to collect what is asked in the usual practice in Primary Care in our environment.

TRIAL REGISTRATION AND CURRENT STATUS

The trial was registered under the title “OUTPATIENT TREATMENT OF EARLY PULMONARY COVID19 WITH CORTICOSTEROIDS AS AN OPPORTUNITY TO MODIFY THE COURSE OF THE DISEASE” with EudraCT number 2020-001622-64, registered on 03/APR/2020. The protocol with code TAC-COVID-19, version 2.0 on date: 16/APRIL/2020 is already approved by the Spanish Drug Agency (AEMPS). The trial is already in the recruitment phase.

ETHICS APPROVAL AND CONSENT TO PARTICIPATE

The protocol was evaluated by the Local Research Ethics Committee of Burgos, Spain, on 17/APR/2020 with the reference number 2295. It was approved on 17/APR/2020.

Patients will be asked for their verbal informed consent with the presence of witnesses, and this will be reflected in the medical record before included in the clinical trial.

REFERENCES

Lee KY, Ito K, Maneechotesuwan K. Inflammation to Pulmonary Diseases. *Mediators Inflamm*. 2016;2016:7401245. doi:10.1155/2016/7401245.

Channappanavar R, Perlman S. Pathogenic human coronavirus infections: causes and consequences of cytokine storm and immunopathology. *Semin Immunopathol*. 2017;39(5):529‐539. doi:10.1007/s00281-017-0629-x

Joynt GM, Wu WK. Understanding COVID-19: what does viral RNA load really mean?. *Lancet Infect Dis*. 2020;20(6):635‐636. doi:10.1016/S1473-3099(20)30237-1

Russell CD, Millar JE, Baillie JK. Clinical evidence does not support corticosteroid treatment for 2019-nCoV lung injury. *Lancet*. 2020;395(10223):473‐475. doi:10.1016/S0140-6736(20)30317-2

Ni YN, Chen G, Sun J, Liang BM, Liang ZA. The effect of corticosteroids on mortality of patients with influenza pneumonia: a systematic review and meta-analysis. *Crit Care*. 2019;23(1):99. Published 2019 Mar 27. doi:10.1186/s13054-019-2395-8

Yamaya M, Nishimura H, Deng X, et al. Inhibitory effects of glycopyrronium, formoterol, and budesonide on coronavirus HCoV-229E replication and cytokine production by primary cultures of human nasal and tracheal epithelial cells. *Respir Investig*. 2020;58(3):155‐168. doi:10.1016/j.resinv.2019.12.005

Matsuyama S., et al. The inhaled corticosteroid ciclesonide blocks coronavirus RNA replication by targeting viral NSP15. bioRxiv preprint doi: https://doi.org/10.1101/2020.03.11.987016.

Spanish Ministry of Health, Consumer Affairs and Social Welfare. Clinical management of COVID-19: inpatient care. Available from: <https://www.mscbs.gob.es/profesionales/saludPublica/ccayes/alertasActual/nCov-China/documentos/Protocolo_manejo_clinico_ah_COVID-19.pdf> Last accessed June 10, 2020
